# Identification of plasma protein markers of allergic disease risk: a mendelian randomization approach to proteomic analysis

**DOI:** 10.1186/s12864-024-10412-0

**Published:** 2024-05-22

**Authors:** Ziqin Cao, Qiangxiang Li, Yajia Li, Jianhuang Wu

**Affiliations:** 1 Department of Spine Surgery and Orthopaedics, Xiangya Hospital, Central South University, Xiangya Road 87, Changsha, 410000 China; 2 National Clinical Research Center for Geriatric Disorders, Central South University, Changsha, China; 3 Department of Dermatology, Xiangya Hospital, Central South University, Changsha, Hunan 410011 China

**Keywords:** Proteome, Plasma protein, Mendelian randomization, Allergy, Atopic dermatitis, Allergic asthma, Allergic rhinitis

## Abstract

**Background:**

While numerous allergy-related biomarkers and targeted treatment strategies have been developed and employed, there are still signifcant limitations and challenges in the early diagnosis and targeted treatment for allegic diseases. Our study aims to identify circulating proteins causally associated with allergic disease-related traits through Mendelian randomization (MR)-based analytical framework.

**Methods:**

Large-scale cis-MR was employed to estimate the effects of thousands of plasma proteins on five main allergic diseases. Additional analyses including MR Steiger analyzing and Bayesian colocalisation, were performed to test the robustness of the associations; These findings were further validated utilizing meta-analytical methods in the replication analysis. Both proteome- and transcriptome-wide association studies approach was applied, and then, a protein-protein interaction was conducted to examine the interplay between the identified proteins and the targets of existing medications.

**Results:**

Eleven plasma proteins were identified with links to atopic asthma (AA), atopic dermatitis (AD), and allergic rhinitis (AR). Subsequently, these proteins were classified into four distinct target groups, with a focus on tier 1 and 2 targets due to their higher potential to become drug targets. MR analysis and extra validation revealed STAT6 and TNFRSF6B to be Tier 1 and IL1RL2 and IL6R to be Tier 2 proteins with the potential for AA treatment. Two Tier 1 proteins, CRAT and TNFRSF6B, and five Tier 2 proteins, ERBB3, IL6R, MMP12, ICAM1, and IL1RL2, were linked to AD, and three Tier 2 proteins, MANF, STAT6, and TNFSF8, to AR.

**Conclusion:**

Eleven Tier 1 and 2 protein targets that are promising drug target candidates were identified for AA, AD, and AR, which influence the development of allergic diseases and expose new diagnostic and therapeutic targets.

**Supplementary Information:**

The online version contains supplementary material available at 10.1186/s12864-024-10412-0.

## Introduction

Allergic diseases arise from inappropriate initiation of type 2 immune responses against innocuous environmental antigens and include disorders such as atopic dermatitis (AD), allergic asthma (AA), allergic rhinitis (AR), allergic conjunctivitis (AC) and allergic urticaria (AU) [1, 2]. The development of AD in early life, followed by other allergies, such as asthma, has been described as the atopic march [3, 4]. Allergic disease incidence has increased over the past 3 decades to affect an estimated 433 million people worldwide and exert a considerable economic and social burden [5, 6]. A combination of genetics, exposure to environmental allergens and irritants, microbial interactions, and abnormal immune responses contribute to inflammation and the atopic march [7, 8]. However, allergic disease pathogenesis is complex and poorly understood with limited drug options to tackle the recurrent and potentially life-long nature. These observations illustrate the need for further mechanistic studies and identification of drug targets.

The human plasma proteome participates in inter-tissue communications via metabolic, signaling, and physiological pathways and includes potential drug targets [9–13]. The link between levels of specific plasma proteins and allergic disease risk has been reported previously but residual confounders and reverse causation mean that observational studies do not demonstrate causality [14, 15]. Mendelian randomization (MR) uses genetic variation as an instrumental variable (IV) to explore the causal effect of exposure on outcomes. The impact of genes or proteins is difficult to interpret from isolated GWAS outcomes due to the occurrence of single nucleotide polymorphisms (SNPs). However, proteome-wide association studies (PWAS) give a clearer picture of the influence of proteins on disease [16]. In addition, functional summary-based imputation software allows transcriptome-wide association studies (TWAS) to assess the impact of whole blood protein-coding gene expression on allergic disease risk [17].

**Fig. 1:**
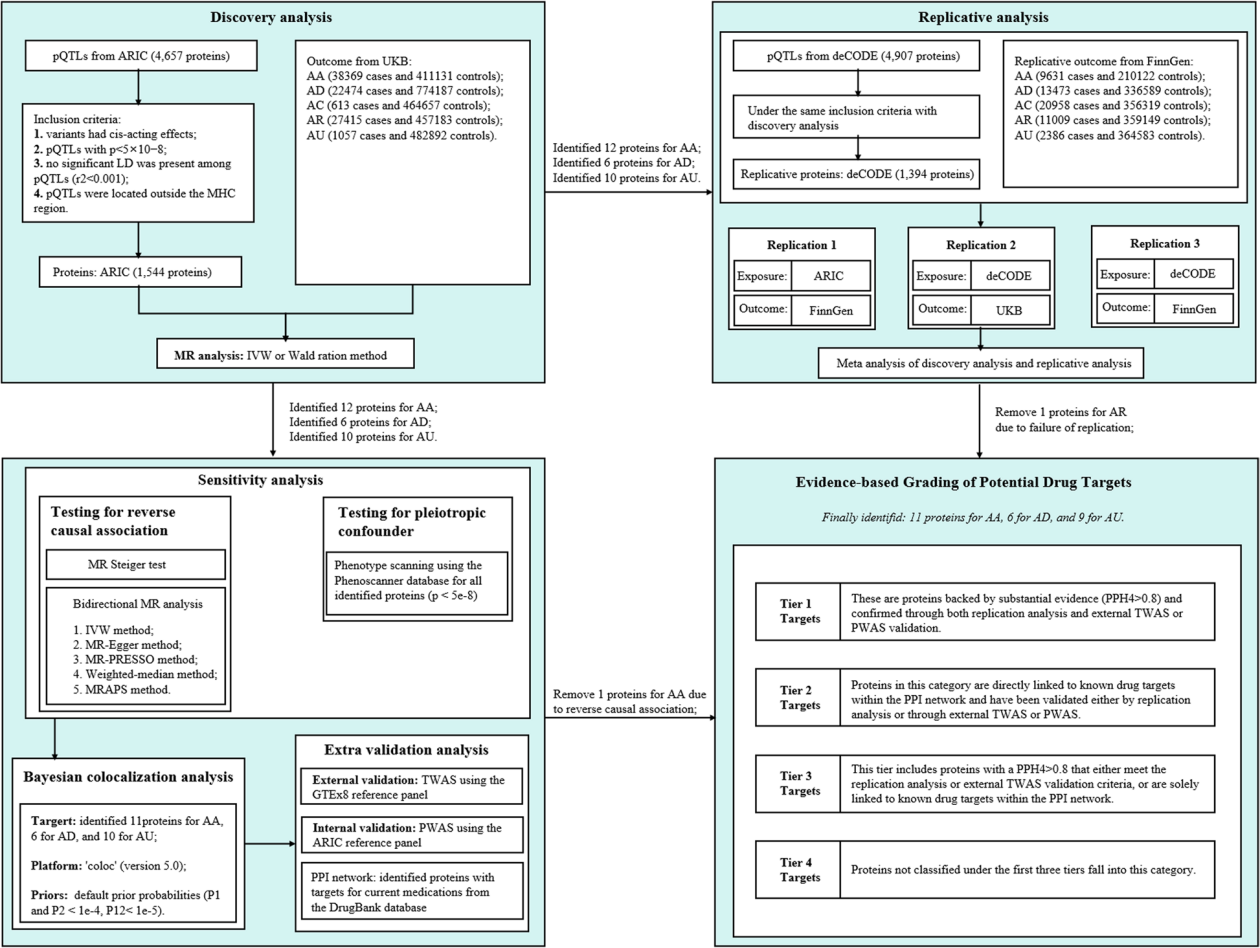
Flowchart of evidence-based grading of potential drug targets from plasma protein on allergic diseases. AA: Allergic asthma; AC: Allergic conjunctivitis; AD: Atopic dermatitis; AR: Allergic rhinitis; AU: Allergic urticaria; PPI: Protein–protein interaction; pQTL: Protein quantitative trait loci; PPH4: posterior probability of hypothesis 4; PWAS: proteome-wide association studies; TWAS: transcriptome-wide association studies

The current study used a PWAS approach, combining GWAS data with protein quantitative trait locus (pQTL) and TWAS with expression quantitative trait loci (eQTL), to investigate potential proteinaceous diagnostic and therapeutic targets in allergic disease. Bayesian colocalization analysis and phenotype scanning were conducted to verify causality between candidate proteins and disease pathogenesis. A protein-protein interaction (PPI) network was constructed and pathway enrichment was performed to illuminate potential mechanisms. The detailed research flowchart is presented in Fig. 1.

## Materials and methods

### Data sources and selection of IVs

Whole blood pQTL data concerning 4,657 proteins derived from 7,213 participants of European descent in the ARIC study was analyzed [18]. SomaScan technology on the v.4.1 platform (SomaLogic) was used for proteomic profiling. pQTLs were filtered according to the following criteria: (1) variants had cis-acting effects, defined as being situated within a 1 Mb region up- or downstream of the gene encoding the plasma protein; (2) pQTLs met the genome-wide significance threshold of *p* < 5 × 10 − 8; (3) no significant linkage disequilibrium (LD) was present among pQTLs (r2 < 0.001); (4) pQTLs were located outside the major histocompatibility complex (MHC) region (chr6, 26-34 Mb). The analysis was repeated using pQTL data from 35,559 Icelandic individuals, including 4,907 plasma proteins (Icelandic Cancer Project and deCODE genetics, Reykjavík, Iceland) on SomaScan v 4.0 platform [19].

Data for individual allergic diseases, including 38,369 cases and 411,131 controls for AA, 613 cases and 464,657 controls for AC, and 1,057 cases and 482,892 controls for AU were obtained from a UKB cohort GWAS study [20]. A UKB cohort from a trans-biobank meta-analysis study [21] yielded data for 22,474 cases of AD and 774,187 controls. Data on 27,415 cases of AR and 457,183 controls came from a separate GWAS study [22]. Summary statistics for the allergic diseases under scrutiny were obtained from the FinnGen Biobank Analysis Consortium database (https://finngen.gitbook.io/documentation/). Allergic diseases were diagnosed according to ICD-10 (International Classification of Diseases) criteria. Data sources are presented in detail in S. Table 1.

### Statistical analysis

#### Mendelian randomization analysis

MR analysis was performed using R software (version 4.1.3) and the TwoSampleMR package (version 0.5.6) [23]. Effect estimates of plasma proteins with a single SNP were generated using the Wald ratio [24] and of those with two or more SNPs by the Inverse Variance Weighted (IVW) method [25]. A false discovery rate (FDR, *p* < 0.05) correction was applied to account for multiple comparisons in validating MR results [26]. Results are expressed as odds ratios per standard deviation increase in genetically determined plasma protein levels. Considering the genetic representativeness of the results, the ARIC/UKB combination, which includes a mixed European genetic background, was chose as the discovery analysis.

#### Sensitivity analysis

Reverse causality between the proteins identified by discovery analysis and allergic diseases was evaluated by bidirectional MR analysis and MR Steiger test [26]. Four additional methods were employed in bidirectional MR analysis to assess the IV validity focusing on the three IV principles: strong association with exposure, direct influence on the outcome through exposure, and no association with outcome confounders. Violations of these principles could compromise the accuracy of the results. MR-Egger regression slope and intercept were used to estimate pleiotropy across IVs to give an adjusted estimate independent of IV validity [27, 28]. MR-PRESSO was used to identify outliers causing significant pleiotropy and heterogeneity to give a corrected causal effect assessment [29]. The weighted-median method enabled consistent inference even when over 50% of IVs were valid [30]. MR-Robust Adjusted Profile Score (MRAPS) increased statistical power to give robust estimates in the presence of weak instrumental bias and horizontal pleiotropy [31]. By default, IVW results are preferred [32], but we turn to MR-Egger when significant pleiotropy is detected by the MR-Egger pleiotropy test. If the MR-PRESSO global test identifies significant outliers, we prioritize results corrected by MR-PRESSO. A Bonferroni correction was applied to address multiple comparison errors and a corrected value of *p* < 0.01 was considered to indicate significance in reverse MR. Plasma proteins that appeared significant during both MR Steiger and reverse MR analysis, suggestive of reverse causality, were excluded from further analysis.

Potential links between all identified proteins and confounders were investigated via phenotype scanning using the Phenoscanner database [33] with a genome-wide significance threshold of *p* < 5 × 10^−8^. pQTLs linked to known allergic disease factors, indicative of pleiotropic effects, were interpreted with caution.

Then, proteins identified by discovery analysis were selected for replication analysis in further multi-center MR studies. Validation alternated between the two sets of pQTL and outcome data from FinnGen and UKB (including three validation sets: ARIC/FinnGen, deCODE/UKB, and deCODE/FinnGen), using genome-wide significant SNPs as genetic instruments. The stability of causal associations was evaluated through meta-analysis with a value of I²>50% indicating significant heterogeneity, necessitating a random-effects model [34].

#### Bayesian colocalization analysis

Colocalization analysis [35] was employed to determine whether a specific genetic variant influenced both an exposure factor and an outcome by modulating gene expression at common loci. Bayesian analysis to calculate the posterior probability of a shared causal variant influencing two traits was performed using the R package ‘coloc’ (version 5.0, available at https://github.com/chr1swallace/coloc) with a default prior probabilities: a prior probability of 1e-4 for any single SNP being associated with each trait (P1 and P2) and a prior probability of 1e-5 for a SNP being associated with both traits (P12) [36]. Assuming a single causal variant, four hypotheses were considered: H0: no causal variants for either trait; H1: a causal variant for the first trait only; H2: a causal variant for the second trait only; H3: separate causal variants for both traits and H4: a shared causal variant for both traits [37]. Significant colocalization was inferred when the posterior probability of H4 was > 0.8, implying strong evidence of a shared causal influence [38].

#### Extra validation analysis

Considering that gene expression and protein synthesis are influenced by numerous factors beyond simple genetic processes, we conducted extra validation analyses to verify the results of our discovery analysis at both the tissue and protein levels. This validation was performed using both TWAS and PWAS methods predictive of gene influence on phenotype generated by Functional Summary-Based Imputation (FUSION) software (available at http://gusevlab.org/projects/fusion), based on the utility of GWAS summary statistics to indicate associations between GWAS phenotypes and functional phenotypes. TWAS indicated the association of protein-coding genes with allergic disease risk at the tissue level and was used as external validation analysis which utilized the pre-computed eQTL reference panel for target proteins derived from the GTEx8 (Genotype-Tissue Expression version 8) database. Likewise, the PWAS served as an internal validation analysis that integrated the GWAS summary statistics and the pre-computed plasma proteome reference weight also from the ARIC study [17] to calculate the genetic impact on allergic disease. Thus, the impact of significant SNPs from the GWAS on protein abundance could be evaluated and candidate genes linked to allergic disease that regulate plasma protein levels identified. An similar FDR corrected p value < 0.05 was the threshold of significance in the extra validation analysis.

A PPI network was constructed using the Search Tool for the Retrieval of Interacting Genes (STRING) database (version 11.5) [39] with a minimum required interaction score (IAS) threshold of 0.4 to indicate interactions among identified proteins and pre-existing anti-allergy drug targets [40]. Information on anti-allergy drug targets was sourced from the DrugBank database.

#### Evidence-based grading of potential drug targets

Proteins were graded according to the criteria of Feihong Ren [41].

- Tier 1 Targets: substantial evidence (PPH4 > 0.8) for drug targeting, confirmed by replication analysis and extra TWAS or PWAS validation.
- Tier 2 Targets: direct linkage to known drug targets within the PPI network, validated either by replication analysis or extra TWAS or PWAS.
- Tier 3 Targets: proteins with a PPH4 > 0.8, validated by either replication analysis or extra TWAS criteria or linked to known drug targets within the PPI network.
- Tier 4 Targets: Proteins not classified under the first three tiers.

## Results

### Discovery MR analysis

**Fig. 2:**
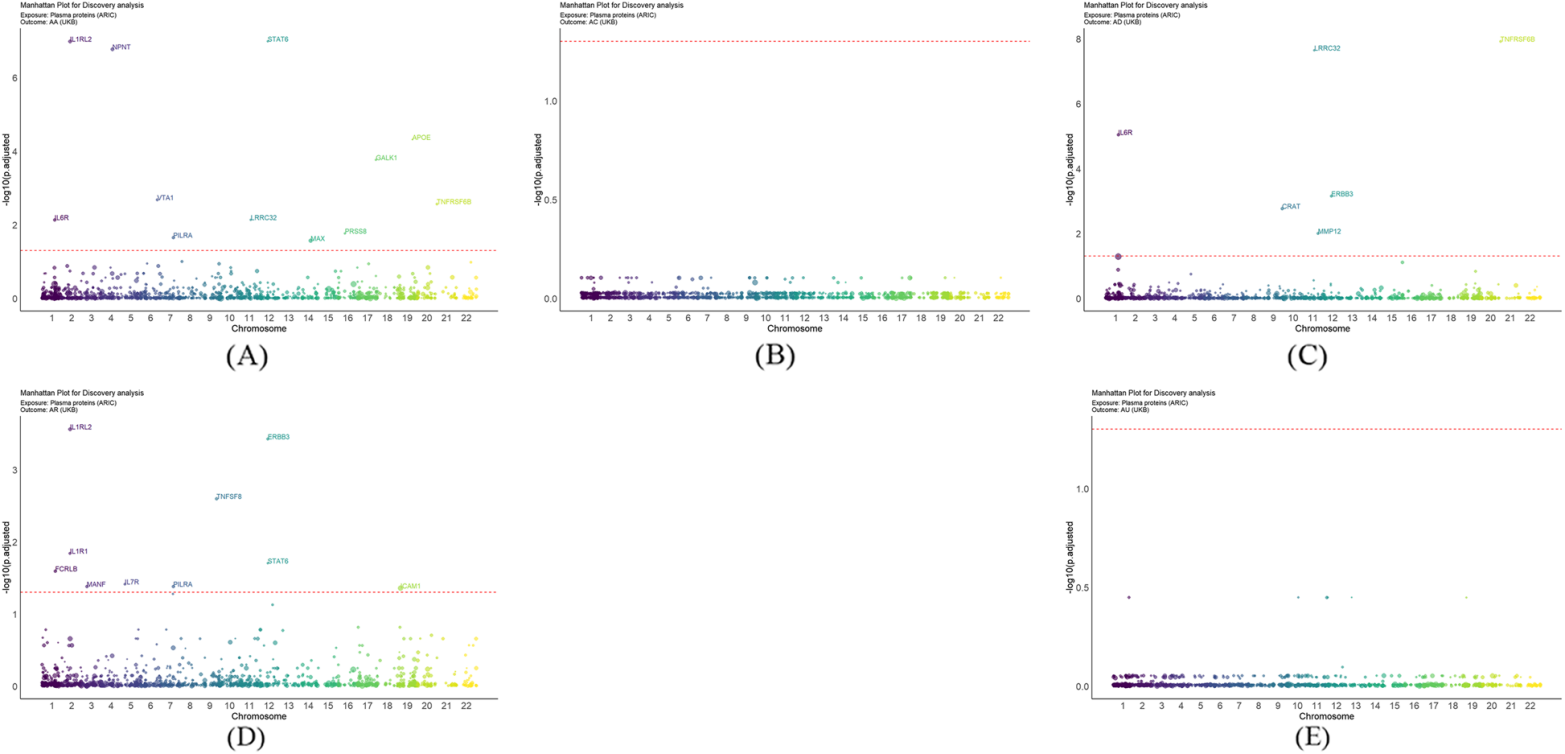
Manhattan plot illustrating the the chromosomal distribution of identified plasma proteins for allergic diseases. The standard line in the plot represents the threshold of FDR *P* = 0.05. **A** Allergic asthma; **B** Allergic conjunctivitis; **C** Atopic dermatitis; **D** Allergic rhinitis; **E** Allergic urticaria

**Fig. 3:**
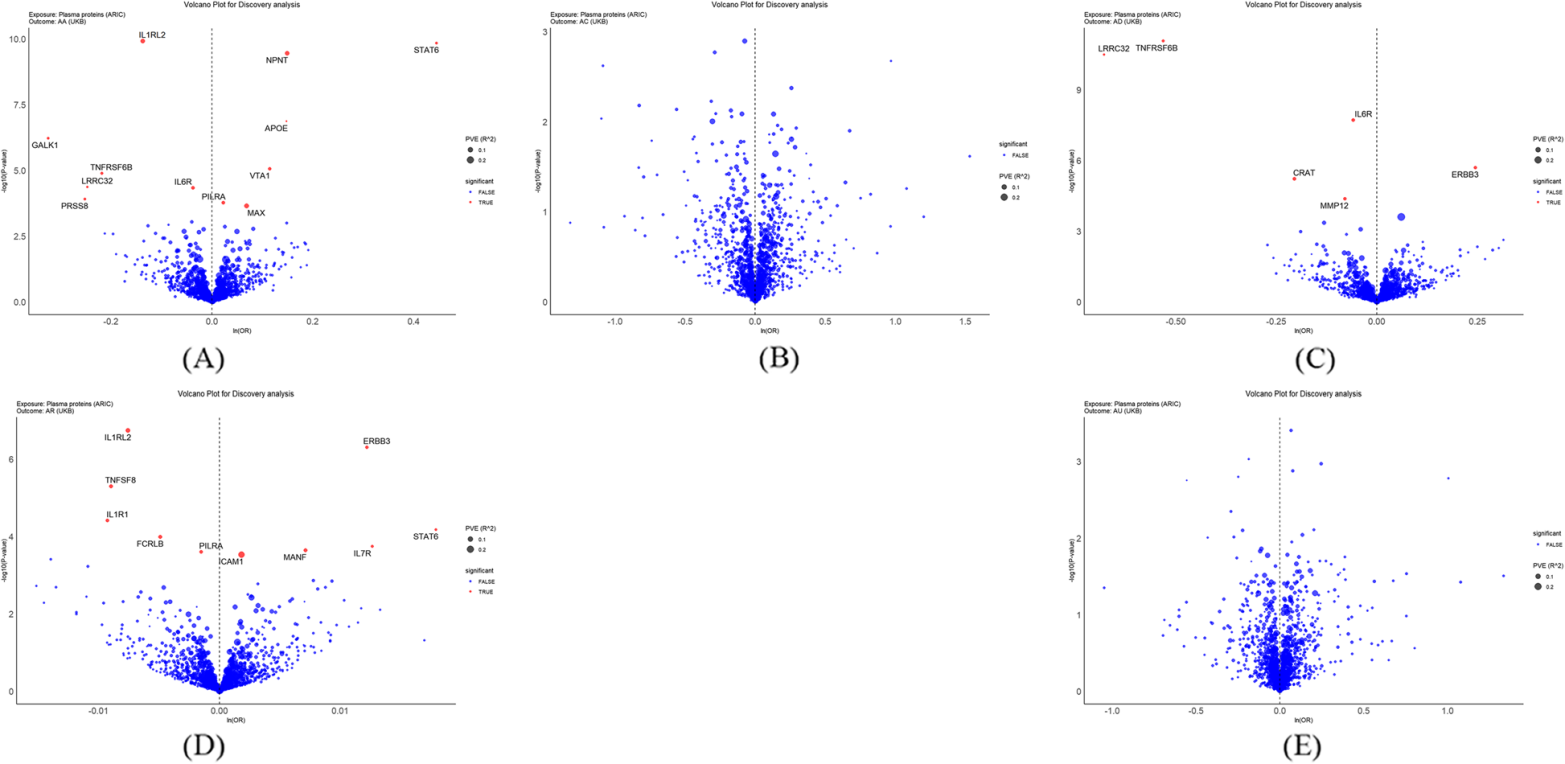
Volcano plots of the MR results from the discovery analysis, displaying the associations between 1,544 proteins from ARIC and the risk of allergic diseases. The increased OR for allergic diseases risk is represented as increments in SD of plasma protein levels. Red dots indicate significant proteins. ‘ln’ refers to the natural logarithm; ‘PVE’ stands for the proportion of variance explained. **A** Allergic asthma; **B** Allergic conjunctivitis; **C** Atopic dermatitis; **D** Allergic rhinitis; **E** Allergic urticaria

**Fig. 4:**
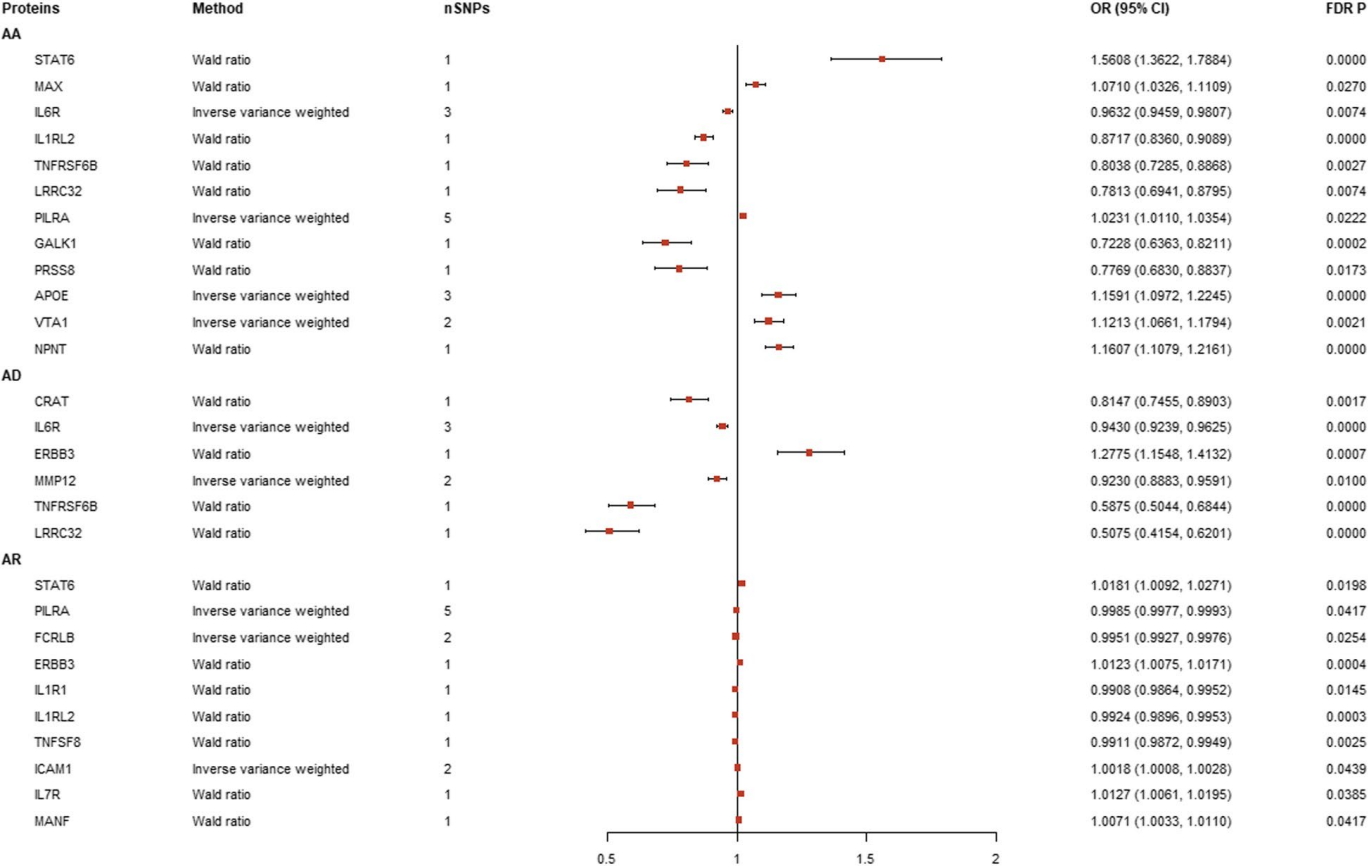
Forest plot of MR results from the discovery analysis. AA: Allergic asthma; AD: Atopic dermatitis; AR: Allergic rhinitis

A total of 1,544 proteins associated with 2,810 SNPs (S. Table 2) were identified during initial discovery analysis. Chromosomal locations are shown by Manhattan plots (Fig. 2). After FDR adjustment, 12 proteins were linked to AA, 6 to AD and 10 to AR but no proteins associated with AC or AU were found (Fig. 3). APOE (OR: 1.1591, 95% CI: 1.0972, 1.2245), MAX (OR: 1.0710, CI: 1.0326, 1.1109), NPNT (OR: 1.1607, CI: 1.1079, 1.2161), PILRA (OR: 1.0231, CI: 1.0110, 1.0354), STAT6 (OR: 1.5608, CI: 1.3622, 1.7884) and VTA1 (OR: 1.1213, CI: 1.0661, 1.1794) were associated with increased AA risk and GALK1 (OR: 0.7228, CI: 0.6363, 0.8211), IL1RL2 (OR: 0.8717, CI: 0.8360, 0.9089), IL6R (OR: 0.9632, CI: 0.9459, 0.9807), LRRC32 (OR: 0.7813, CI: 0.6941, 0.8795), PRSS8 (OR: 0.7769; CI: 0.6830, 0.8837) and TNFRSF6B (OR: 0.8038, CI: 0.7285, 0.8868) with decreased risk. ERBB3 (OR: 1.0123, CI: 1.0075, 1.0171), ICAM1 (OR: 1.0018, CI: 1.0008, 1.0028), IL7R (OR: 1.0127, CI: 1.0061, 1.0195), MANF (OR: 1.0071, CI: 1.0033, 1.0110) and STAT6 (OR: 1.0181; CI: 1.0092, 1.0271) were associated with increased risk of AR and FCRLB (OR: 0.9951, CI: 0.9927 to 0.9976), IL1R1 (OR: 0.9908, CI: 0.9864, 0.9952), IL1RL2 (OR: 0.9924, CI: 0.9896, 0.9953), PILRA (OR: 0.9985, CI: 0.9977, 0.9993) and TNFSF8 (OR: 0.9911, CI: 0.9872, 0.9949) with decreased risk. All proteins, with the exception of ERBB3 (OR: 1.2775, CI: 1.1548, 1.4132), were linked to lower AD risk. Results are visualized in forest plots (Fig. 4).

### Replicative MR and meta-analysis

A total of 1,394 novel proteins with 4,144 SNPs were identified from deCode data by a similar IV selection process (S. Table 3) and used to validate the findings of discovery analysis from the FinnGen datasets (Table 1). Replication analysis using deCode data showed that GALK1, IL1RL2, and TNFRSF6B failed to replicate for AA, as did VTA1 and TNFRSF6B for AD and FCRLB, IL1RL2 and MANF for AR. IL7R could not be replicated during any iteration and was excluded from further analysis. Meta-analysis showed robust associations for other proteins with AA except APOE (*p* = 0.8196; OR: 1.0152, 95% CI: 0.8918, 1.1557) and LRRC32 (*p* = 0.1864; OR: 0.8048, CI: 0.5832, 1.1107, S. Figure 1). Significant associations also remained for proteins with AD, apart from LRRC32 (*p* = 0.0990; OR: 0.6015, 95% CI: 0.3288, 1.1002, S. Figure 2). Only STAT6 and PILRA retained a significant association with AR following post-replication analysis (S. Figure 3). IL7R in AR was excluded from further analysis due to the failure of replication.

**Table 1:** Detailes of the discovery analysis and replicative validations

Proteins	Uniprot ID	OR (95%CI)	FDR P	OR (95%CI)	FDR P	OR (95%CI)	FDR P	OR (95%CI)	FDR P	I2	Models	OR (95%CI)	P	Outcome
		ARIC and UKB		ARIC and FinnGen		Decode and UKB		Decode and FinnGen		Meta-analysis				
APOE	P02649	1.1591 (1.0972, 1.2245)	4.83E-05	1.1039 (0.9955, 1.2241)	6.25E-01	0.9144 (0.8578, 0.9748)	2.84E-01	0.8934 (0.7844, 1.0176)	6.68E-01	91.90%	Random-effect	1.0152 (0.8918, 1.1557)	8.20E-01	AA
GALK1	P51570	0.7228 (0.6363, 0.8211)	1.71E-04	0.5958 (0.4337, 0.8184)	1.18E-01	NA	NA	NA	NA	18.43%	Fixed-effect	0.7036 (0.6251, 0.7920)	5.80E-09	
IL1RL2	Q9HB29	0.8717 (0.8360, 0.9089)	1.02E-07	0.7943 (0.7252, 0.8700)	4.75E-04	NA	NA	NA	NA	69.78%	Random-effect	0.8397 (0.7680, 0.9182)	1.27E-04	
IL6R	P08887	0.9632 (0.9459, 0.9807)	7.38E-03	0.9417 (0.9132, 0.9711)	2.84E-02	0.9683 (0.9243, 1.0145)	7.83E-01	1.0119 (0.9466, 1.0818)	9.51E-01	27.47%	Fixed-effect	0.9611 (0.9473, 0.9751)	6.93E-08	
LRRC32	Q14392	0.7813 (0.6941, 0.8795)	7.38E-03	0.5497 (0.4057, 0.7448)	2.84E-02	1.1925 (0.9403, 1.5123)	7.64E-01	0.7919 (0.4868, 1.2882)	9.35E-01	82.15%	Random-effect	0.8048 (0.5832, 1.1107)	1.86E-01	
MAX	P61244	1.0710 (1.0326, 1.1109)	2.70E-02	1.1617 (1.0769, 1.2531)	2.84E-02	1.1605 (1.0715, 1.2568)	3.98E-02	1.3949 (1.1820, 1.6461)	2.71E-02	77.83%	Random-effect	1.1637 (1.0666, 1.2696)	6.49E-04	
NPNT	Q6UXI9	1.1607 (1.1079, 1.2161)	1.69E-07	1.1261 (1.0326, 1.2279)	2.45E-01	1.3415 (1.2238, 1.4705)	2.23E-07	1.2637 (1.0654, 1.4989)	3.11E-01	68.91%	Random-effect	1.2096 (1.1139, 1.3135)	6.04E-06	
PILRA	Q9UKJ1	1.0231 (1.0110, 1.0354)	2.22E-02	1.0055 (0.9797, 1.0320)	9.83E-01	1.0110 (0.9906, 1.0319)	8.76E-01	1.0164 (0.9711, 1.0639)	9.39E-01	0.00%	Fixed-effect	1.0180 (1.0085, 1.0275)	1.94E-04	
PRSS8	Q16651	0.7769 (0.6830, 0.8837)	1.73E-02	0.7719 (0.6011, 0.9911)	5.53E-01	0.5990 (0.4561, 0.7866)	3.98E-02	0.5975 (0.3538, 1.0089)	6.05E-01	16.93%	Fixed-effect	0.7398 (0.6671, 0.8205)	1.15E-08	
STAT6	P42226	1.5608 (1.3622, 1.7884)	1.02E-07	1.7634 (1.3338, 2.3314)	2.84E-02	1.0315 (0.8989, 1.1838)	9.79E-01	1.1330 (0.8091, 1.5866)	9.39E-01	86.95%	Random-effect	1.3364 (1.0367, 1.7228)	2.52E-02	
TNFRSF6B	O95407	0.8038 (0.7285, 0.8868)	2.69E-03	0.6561 (0.5222, 0.8243)	4.97E-02	NA	NA	NA	NA	61.00%	Random-effect	0.7462 (0.6161, 0.9039)	2.76E-03	
VTA1	Q06263	1.1213 (1.0661, 1.1794)	2.09E-03	1.1228 (0.9921, 1.2708)	6.27E-01	1.9728 (1.4726, 2.6428)	2.19E-03	1.6285 (0.8949, 2.9632)	7.23E-01	80.45%	Random-effect	1.3524 (1.0139, 1.8039)	4.00E-02	
CRAT	P43155	0.8147 (0.7455, 0.8903)	1.70E-03	0.8067 (0.7184, 0.9058)	3.40E-02	NA	NA	NA	NA	0.00%	Fixed-effect	0.8104 (0.7630, 0.8606)	7.54E-12	AD
ERBB3	P21860	1.2775 (1.1548, 1.4132)	7.03E-04	1.2871 (1.1333, 1.4617)	1.94E-02	1.5768 (1.3069, 1.9024)	2.43E-03	1.5989 (1.2620, 2.0256)	3.63E-02	39.45%	Fixed-effect	1.3306 (1.2517, 1.4144)	5.06E-20	
IL6R	P08887	0.9430 (0.9239, 0.9625)	9.14E-06	0.9363 (0.8546, 1.0258)	8.69E-01	0.9494 (0.9261, 0.9732)	1.62E-02	0.9423 (0.9136, 0.9720)	3.63E-02	0.00%	Fixed-effect	0.9445 (0.9316, 0.9576)	4.00E-16	
LRRC32	Q14392	0.5075 (0.4154, 0.6201)	2.28E-08	0.3207 (0.2462, 0.4176)	2.23E-14	1.1788 (0.8348, 1.6645)	9.90E-01	1.3638 (0.9030, 2.0597)	7.65E-01	94.14%	Random-effect	0.6015 (0.3288, 1.1002)	9.90E-02	
MMP12	P39900	0.9230 (0.8883, 0.9591)	9.98E-03	0.8911 (0.8539, 0.9300)	5.38E-05	0.9255 (0.8890, 0.9634)	4.86E-02	0.9051 (0.8636, 0.9485)	3.48E-02	0.00%	Fixed-effect	0.9083 (0.8914, 0.9254)	7.42E-24	
TNFRSF6B	O95407	0.5875 (0.5044, 0.6844)	1.20E-08	0.4312 (0.3547, 0.5243)	2.23E-14	NA	NA	NA	NA	76.92%	Random-effect	0.4819 (0.3909, 0.5942)	8.49E-12	
ERBB3	P21860	1.0123 (1.0075, 1.0171)	3.72E-04	0.9825 (0.8542, 1.1300)	9.99E-01	0.9776 (0.9689, 0.9862)	3.13E-04	0.9677 (0.7461, 1.2551)	9.90E-01	93.55%	Random-effect	0.9941 (0.9640, 1.0251)	7.05E-01	AR
FCRLB	Q6BAA4	0.9951 (0.9927, 0.9976)	2.54E-02	0.9206 (0.8547, 0.9915)	6.16E-01	NA	NA	NA	NA	76.34%	Random-effect	0.9659 (0.8969, 1.0403)	3.60E-01	
ICAM1	Q5NKV9	1.0018 (1.0008, 1.0028)	4.39E-02	1.0017 (0.9734, 1.0309)	9.99E-01	0.9982 (0.9964, 1.0000)	5.95E-01	1.0930 (1.0448, 1.1435)	2.27E-02	88.78%	Random-effect	1.0182 (0.9813, 1.0565)	3.39E-01	
IL1R1	P14778	0.9908 (0.9864, 0.9952)	1.45E-02	0.8672 (0.7585, 0.9916)	6.41E-01	1.0117 (1.0063, 1.0171)	5.65E-03	0.8750 (0.7436, 1.0298)	7.87E-01	92.85%	Random-effect	0.9667 (0.9068, 1.0305)	2.99E-01	
IL1RL2	Q9HB29	0.9924 (0.9896, 0.9953)	2.71E-04	0.8438 (0.7757, 0.9179)	5.15E-02	NA	NA	NA	NA	92.99%	Random-effect	0.9203 (0.7853, 1.0785)	3.05E-01	
IL7R	P16871	1.0127 (1.0061, 1.0195)	3.85E-02	NA	NA	NA	NA	NA	NA	NA	NA	NA	NA	
MANF	P55145	1.0071 (1.0033, 1.0110)	4.17E-02	1.1749 (1.0514, 1.3128)	3.82E-01	NA	NA	NA	NA	86.46%	Random-effect	1.0765 (0.9270, 1.2502)	3.34E-01	
PILRA	Q9UKJ1	0.9985 (0.9977, 0.9993)	4.17E-02	1.0053 (0.9799, 1.0315)	9.99E-01	0.9983 (0.9970, 0.9996)	3.81E-01	1.0092 (0.9673, 1.0530)	9.78E-01	0.00%	Fixed-effect	0.9984 (0.9977, 0.9991)	1.10E-05	
STAT6	P42226	1.0181 (1.0092, 1.0271)	1.98E-02	1.5063 (1.1623, 1.9523)	2.51E-01	1.0152 (1.0035, 1.0269)	3.81E-01	1.2486 (0.9088, 1.7155)	8.43E-01	71.58%	Random-effect	1.0174 (1.0103, 1.0245)	1.36E-06	
TNFSF8	P32971	0.9911 (0.9872, 0.9949)	2.51E-03	1.0534 (0.9425, 1.1773)	9.73E-01	1.0120 (0.9984, 1.0259)	6.60E-01	1.1328 (0.7257, 1.7683)	9.71E-01	69.58%	Random-effect	1.0023 (0.9822, 1.0228)	8.23E-01	

*OR* Odds ratio, *FDR* False discovery rate, *AA* Allergic asthma, *AD* Atopic dermatitis

### Sensitivity analysis

Steiger filtering confirmed causal relationship directionality with only the relationship of APOE to AA failing to pass the Steiger test (R2xy = 3.36 × 10-6; *p* = 0.878). Further bidirectional MR analysis confirmed the significant reverse causal association between AA and APOE (OR: 2.5883, CI: 1.8731, 3.5766, S. Table 4). Consequently, AA was excluded from further analysis.

 Phenotype scanning indicated potential pleiotropic effects with APOE, GALK1, ICAM1, MAX, PRSS8, and VTA1 being associated with body mass and blood lipid levels. APOE and PILRA have been previously linked to diabetes, a condition that may be comorbid with allergic diseases. Furthermore, APOE has been associated with the phenotype of maternal diabetes, a condition that correlates with a higher risk of allergic disease in offspring. In addition, ERBB3, IL1R1, IL1RL2, IL6R, LRRC32, STAT6, and TNFRSF6B have been strongly linked to allergic diseases, such as asthma and rhinitis (S. Table 5).

#### Colocalization analysis and extra validation

Colocalization analysis was performed to assess any shared genetic signals between the proteins and AA or AD with a predefined threshold of PPH4 ≥ 0.8. The majority of proteins were found to colocalize with AA, except for APOE (PPH4 = 1.54%), IL1RL2 (PPH4 = 0.00%), MAX (PPH4 = 75.70%), and PILRA (PPH4 = 0.19%). Only LRRC32 (PPH4 = 27.4%) failed to show colocalization with AD. However, substantial colocalization with AR was only observed for ERBB3 (PPH4 = 98.40%) and TNFSF8 (PPH4 = 88.00%, S. Table 6 and S. Figures 4–6).

 Two different sets of reference panels from the GTEx database were used for TWAS of AD skin tissue and AA or AR respiratory tract tissue. IL1RL2 and NPNT were mismatched and only STAT6 (Ptwas = 0.0011), TNFRSF6B (Ptwas = 0.0058), and VTA1 (Ptwas = 0.0052) showed significant association with AA among the remaining ten proteins. Similarly, ICAM1, IL1RL2, TNFSF8, and FCRLB were absent from the database but significant associations with AR were found for IL7R (Ptwas = 0.0023), ERBB3 (Ptwas = 0.0444), MANF (Ptwas = 0.0122) and PILRA (Ptwas = 0.0486). CRAT (Ptwas = 0.0000), TNFRSF6B (Ptwas = 0.0002), and LRRC32 (Ptwas = 0.0000) levels correlated with AD. PWAS was performed on the reference panel from the ARIC study and 4 proteins, STAT6, TNFRSF6B, GALK1, and CRAT, were excluded due to mismatches. Other proteins maintained stable associations with the exception of APOE (ppwas = 0.4915), NPNT (ppwas = 0.1310), and LRRC32 (ppwas = 0.0831) for AA and IL1R1 (ppwas = 0.1330) for AR (Table 2 and S. Figure 7).

**Table 2:** Assessment of druggability and evidence grading

Proteins	Discovery analysis		Meta-analysis of Replication analysis			Colocalization analysis		External validation			Steigier direction test		PPI network	Levels of Evidence
	OR (95%)	FDR P	OR (95%)	FDR P	Pass/Fail	PPH4	Pass/Fail	TWAS Z, FDR P	PWAS Z, FDR P	Pass/Fail	P	Pass/Fail	Pass/Fail	
AA														
APOE	1.1591 (1.0972, 1.2245)	4.83E-05	1.0152 (0.8918, 1.1557)	8.20E-01	Fail	1.5%	Fail	-1.0730, 0.7651	-0.6880, 0.9665	Fail	8.78E-01	Fail	Pass	Excluded
STAT6	1.5608 (1.3622, 1.7884)	1.02E-07	1.3364 (1.0367, 1.7228)	2.52E-02	Pass	100.0%	Pass	3.2652, 0.0432	NA	Pass	4.67E-10	Pass	Pass	Tier 1
TNFRSF6B	0.8038 (0.7285, 0.8868)	2.69E-03	0.7462 (0.6161, 0.9039)	2.76E-03	Pass	99.4%	Pass	-2.7590, 0.1572	NA	Fail	9.80E-16	Pass	Pass	Tier 2
IL1RL2	0.8717 (0.8360, 0.9089)	1.02E-07	0.8397 (0.7680, 0.9182)	1.27E-04	Pass	0.0%	Fail	NA	-4.0486, 0.0013	Pass	6.00E-88	Pass	Pass	Tier 2
IL6R	0.9632 (0.9459, 0.9807)	7.38E-03	0.9611 (0.9473, 0.9751)	6.93E-08	Pass	100.0%	Pass	-1.4100, 0.6611	5.6215, 0.0000	Fail	4.28E-38	Pass	Pass	Tier 3
GALK1	0.7228 (0.6363, 0.8211)	1.71E-04	0.7036 (0.6251, 0.7920)	5.80E-09	Pass	99.9%	Pass	0.7682, 0.8242	NA	Fail	4.62E-11	Pass	Fail	Tier 3
NPNT	1.1607 (1.1079, 1.2161)	1.69E-07	1.2096 (1.1139, 1.3135)	6.04E-06	Pass	100.0%	Pass	NA	1.5119, 0.7020	Fail	2.48E-90	Pass	Fail	Tier 3
PRSS8	0.7769 (0.6830, 0.8837)	1.73E-02	0.7398 (0.6671, 0.8205)	1.15E-08	Pass	84.6%	Pass	-1.4135, 0.5748	3.8410, 0.0055	Fail	1.78E-11	Pass	Fail	Tier 3
VTA1	1.1213 (1.0661, 1.1794)	2.09E-03	1.3524 (1.0139, 1.8039)	4.00E-02	Pass	97.8%	Pass	2.7957, 0.1446	-2.6109, 0.1967	Fail	1.28E-27	Pass	Fail	Tier 3
LRRC32	0.7813 (0.6941, 0.8795)	7.38E-03	0.8048 (0.5832, 1.1107)	1.86E-01	Fail	95.4%	Pass	-1.9494, 0.5000	1.7330, 0.4283	Fail	6.28E-08	Pass	Fail	Tier 4
MAX	1.0710 (1.0326, 1.1109)	2.70E-02	1.1637 (1.0666, 1.2696)	6.49E-04	Pass	75.7%	Fail	-1.6300, 0.6860	-3.6018, 0.0164	Fail	1.22E-115	Pass	Fail	Tier 4
PILRA	1.0231 (1.0110, 1.0354)	2.22E-02	1.0180 (1.0085, 1.0275)	1.94E-04	Pass	0.2%	Fail	-0.7261, 0.9126	2.6438, 0.1449	Fail	3.26E-37	Pass	Fail	Tier 4
AD														
CRAT	0.8147 (0.7455, 0.8903)	1.70E-03	0.8104 (0.7630, 0.8606)	7.54E-12	Pass	99.3%	Pass	4.2501, 0.0046	NA	Pass	1.16E-43	Pass	Fail	Tier 1
TNFRSF6B	0.5875 (0.5044, 0.6844)	1.20E-08	0.4819 (0.3909, 0.5942)	8.49E-12	Pass	98.0%	Pass	-3.6822, 0.0215	NA	Pass	9.77E-16	Pass	Fail	Tier 1
ERBB3	1.2775 (1.1548, 1.4132)	7.03E-04	1.3306 (1.2517, 1.4144)	5.06E-20	Pass	99.7%	Pass	-0.5643, 0.9467	-4.7549, 0.0001	Fail	6.15E-32	Pass	Pass	Tier 2
IL6R	0.9430 (0.9239, 0.9625)	9.14E-06	0.9445 (0.9316, 0.9576)	4.00E-16	Pass	100.0%	Pass	0.9614, 0.8304	5.7955, 0.0000	Fail	4.26E-38	Pass	Pass	Tier 2
MMP12	0.9230 (0.8883, 0.9591)	9.98E-03	0.9083 (0.8914, 0.9254)	7.42E-24	Pass	97.0%	Pass	0.9690, 0.8193	3.8311, 0.0102	Fail	5.37E-34	Pass	Pass	Tier 2
LRRC32	0.5075 (0.4154, 0.6201)	2.28E-08	0.6015 (0.3288, 1.1002)	9.90E-02	Fail	27.4%	Fail	4.3107, 0.0031	3.1004, 0.0515	Fail	6.27E-08	Pass	Fail	Tier 4
AR														
IL7R	1.0127 (1.0061, 1.0195)	3.85E-02	NA	NA	Fail	0.0%	Fail	-3.0526, 0.1818	4.3853, 0.0008	Fail	5.07E-18	Pass	Pass	Excluded
ICAM1	1.0018 (1.0008, 1.0028)	4.39E-02	1.0182 (0.9813, 1.0565)	3.39E-01	Fail	8.1%	Fail	NA	-3.6235, 0.0106	Pass	8.65E-244	Pass	Pass	Tie 2
IL1RL2	0.9924 (0.9896, 0.9953)	2.71E-04	0.9203 (0.7853, 1.0785)	3.05E-01	Fail	0.0%	Fail	NA	-3.1346, 0.0340	Pass	6.05E-88	Pass	Pass	Tie 2
MANF	1.0071 (1.0033, 1.0110)	4.17E-02	1.0765 (0.9270, 1.2502)	3.34E-01	Fail	24.1%	Fail	-2.5070, 0.6411	-3.4132, 0.0482	Fail	1.06E-48	Pass	Pass	Tie 2
STAT6	1.0181 (1.0092, 1.0271)	1.98E-02	1.0174 (1.0103, 1.0245)	1.36E-06	Pass	47.5%	Fail	0.2935, 0.9346	NA	Fail	4.68E-10	Pass	Pass	Tie 2
TNFSF8	0.9911 (0.9872, 0.9949)	2.51E-03	1.0023 (0.9822, 1.0228)	8.23E-01	Fail	88.0%	Pass	NA	4.3603, 0.0007	Pass	1.91E-47	Pass	Pass	Tie 2
ERBB3	1.0123 (1.0075, 1.0171)	3.72E-04	0.9941 (0.9640, 1.0251)	7.05E-01	Fail	98.4%	Pass	-2.0102, 0.5548	-5.0264, 0.0000	Fail	6.21E-32	Pass	Pass	Tie 3
IL1R1	0.9908 (0.9864, 0.9952)	1.45E-02	0.9667 (0.9068, 1.0305)	2.99E-01	Fail	0.0%	Fail	1.3200, 0.8961	1.5022, 0.6188	Fail	1.57E-36	Pass	Pass	Tie 3
FCRLB	0.9951 (0.9927, 0.9976)	2.54E-02	0.9659 (0.8969, 1.0403)	3.60E-01	Fail	12.0%	Fail	NA	-2.5711, 0.1947	Fail	8.03E-49	Pass	Fail	Tier 4
PILRA	0.9985 (0.9977, 0.9993)	4.17E-02	0.9984 (0.9977, 0.9991)	1.10E-05	Pass	0.0%	Fail	1.9719, 0.4586	-2.2443, 0.3289	Fail	3.28E-37	Pass	Fail	Tier 4

Tier 1 Targets: These are proteins backed by substantial evidence (PPH4>0.8) and confirmed through both replication analysis and external TWAS or PWAS validation

Tier 2 Targets: Proteins in this category are directly linked to known drug targets within the PPI network and have been validated either by replication analysis or through external TWAS or PWAS

Tier 3 Targets: This tier includes proteins with a PPH4>0.8 that either meet the replication analysis or external TWAS validation criteria, or are solely linked to known drug targets within the PPI network

Tier 4 Targets: Proteins not classified under the first three tiers fall into this category

*OR* Odds ratio, *FDR* False discovery rate, *MM* Malignant melanoma, *SCC* Squamous-cell carcinoma, *BCC* Basal cell carcinoma

 A PPI network was constructed from DrugBank data to illustrate interactions among anti-allergic drug targets and proteins of interest (S.Table 7). Interactions were found between STAT6, TNFRSF6B, IL1RL2, IL6R, and established drug targets, as evidenced by an IAS greater than 0.4, in the PPI network for AA (S. Figure 8). Similarly, interactions were found for ERBB3, IL6R, and MMP12 in the AD-specific PPI network (S. Figure 9) and for ERBB3, ICAM1, IL1RL2, MANF, STAT6 and TNFSF8 in the AR-specific PPI network (S. Figure 10). Further gene-disease enrichment analysis revealed STAT6 was enriched in various allergic diseases, most notably in AA, with a strength of 1.86 and an FDR P-value of 1.8e-13. IL1RL2, IL6R, ERBB3, ICAM1, IL1R1, and GALK1 were also enriched in categories like immune system disease, autoimmune disease, skin disease, and disease of anatomical entity (S.Table 8). We also conducted KEGG and GO enrichment analyses on genes within the PPI network. With the exception of GALK1, NPNT, PRSS8, VTA1, LRRC32, PILRA, MMP12, MANF, TNFSF8, and FCRLB, genes of other proteins were successfully enriched in multiple pathways, particularly STAT6, IL6R, and IL1R1 proteins, which were involved in several immune and inflammation-related pathways. Except for FCRLB, MANF, and TNFSF8, other proteins were all successfully enriched in specific biological processes (see S.Table 9, and 10 and S. Figure 11).

#### Potential drug targets

Ultimately, 11 proteins for allergic asthma (AA), 6 for atopic dermatitis (AD), and 9 for allergic rhinitis (AR) were evaluated for their potential as drug targets. Among these, STAT6 was identified as excellent Tier 1 potential drug targets for AA as were CRAT and TNFRSF6B for AD. No Tier 1 proteins were identified for AR. Tier 2 proteins included TNFRSF6B, and IL1RL2 for AA; ERBB3, IL6R, MMP12, ICAM1 and IL1RL2 for AD, and ICAM1, IL1RL2, MANF, STAT6 and TNFSF8 for AR. Other proteins were assigned to Tier 3 or below (Table 2).

## Discussion

To the best of our knowledge, this study is the first to scrutinize causal associations between plasma proteins and allergic diseases through the integrated approach of MR, colocalization, Steiger filtering analysis, PAV assessment, eQTLs overlap determination, PPI analysis, pathway enrichment, and drug target evaluation. Eleven plasma proteins were identified with links to AA, AD, and AR. MR analysis and extra validation revealed STAT6 and TNFRSF6B to be Tier 1 and IL1RL2 and IL6R to be Tier 2 proteins with the potential for AA treatment. Two Tier 1 proteins, CRAT and TNFRSF6B, and five Tier 2 proteins, ERBB3, IL6R, MMP12, ICAM1, and IL1RL2, were linked to AD and three Tier 2 proteins, MANF, STAT6, and TNFSF8, to AR.

Many novel biomarkers have been identified by proteomic and metabolomic analyses, although studies on allergic diseases have generally used low throughput methods [42]. Niet-Fontarigo et al. [43] identified 18 potential biomarkers of asthma phenotype and disease severity, including HSPG2 and IGFALS for AA, through a bottom-up/non-targeted proteomics approach. The sample size was modest with 32 healthy controls, 43 AR patients, and 192 asthmatics and failed to distinguish protein biomarkers from pathogenic factors for allergic disease due to the reverse causal characteristics of an observational study.

Several proteins identified by the current study have previously been linked to allergic disease by epidemiological or laboratory studies. Indeed, STAT6 is known to participate in IL-4 signaling and its role in asthma has been extensively studied since both doctor-diagnosed asthma and blood eosinophil counts are known to be linked to STAT6 signaling and the IL-1 receptor family [44]. Baris S et al. [45] have identified a novel inborn error of immunity arising from a STAT6 gain-of-function mutation causing severe allergic dysregulation which is treated by Janus kinase inhibitor therapy. The TNFRSF6 (also called Fas) receptor binds TNFSF6 (FasL) ligands expressed on CD8 + T cells and oligodendrocytes [46–48]. Th1 cells secrete IFN-γ to activate the Fas/FasL system and induce keratinocyte apoptosis in the spongiosis area which may influence the progression of AD. INF-γ and Fas ligand are secreted by activated CD4 + T cells, TNFRSF6 expressed on keratinocytes, and tumor necrosis factor (TNF)-α secreted by both the activated CD4 + T cells and keratinocytes, with cell-mediated cytotoxicity induced by perforin and granzyme B released by CD8 + T cells [46–48].

The IL-36 receptor (IL-36R, IL-1Rrp2, IL1RL2, or IL-1R6) binds all α, β, and γ members of the IL-36 family. IL-36R is expressed in skin, mammary, and mucosal epithelial cell lines and IL-36 mediates intracellular signaling through the IL-36R and IL-1 receptor accessory protein (IL-1RAcP) [49]. IL-36α, IL-36β, and IL-36γ bind IL-36R, form a signal transduction complex with IL-1RAcP, and recruit myeloid differentiation factor 88 to activate mitogen-activated protein kinases mediated by c-Jun N-terminal kinase, extracellular regulated protein kinases 1/2 and the nuclear factor kappa B pathway. The resulting inflammatory mediators have roles in adaptive immunity. IL-36 cytokines released from keratinocytes increase the immunoglobulin (Ig)E production mediated by IL-4 in B cells from AD patients and treatment with anti-IL-36R antibodies decreases IgE and alleviates the disease phenotype [50, 51]. The RNA helicase, DDX5, which regulates the alternative splicing of IL-36R pre-mRNA was found to be down-regulated in keratinocytes from AD patients which promoted the inflammatory response [52]. TNFSF8 (CD30L) is a ligand for the cell surface antigen and marker for Hodgkin lymphoma and related hematologic malignancies, TNFRSF8/CD30. It is considered that inhibition of CD30L/CD30 signaling may constitute a novel biological therapy for AR, since CD30L was shown to amplify Th2 cell effector response in animal models of AR. In vivo treatment with anti-CD30 antibody suppressed AR development and this may be a sufficient target for the treatment of allergic inflammation [53].

Some novel proteins were suggested to have potential causal effects on allergic diseases by the current work. For example, CRAT is a mitochondrial enzyme that transfers acetyl groups between CoA and carnitine during lipid metabolism and links with dermatitis have not been previously reported. CRAT is a key regulator of mitochondrial dysfunction-induced cellular senescence in dermal fibroblasts [54, 55]. Silencing of CRAT is known to cause mitochondrial dysfunction, inflammation and senescence via activation of the cGAS-STING and NF-ĸB pathways [54]. In addition, functional variants of the IL6R have been linked to increased risk of AA and AD but mechanisms remain unclear, although IL-6/soluble IL-6R trans-signaling may affect AD and AA development [56–58]. Genetic variants of ERBB3 have been identified as AD susceptibility factors and serum MMP12 may be an indicator of AD and AR disease pathways [57, 59–61]. Adhesion molecules are known to be involved in T cell homing to skin lesions in AD patients, one example being ICAM-1 which is highly expressed and may have a pathogenic role [62, 63]. Lastly, little attention has been paid to MANF, although the protein is measurable in serum and reflective of extracellular biomarkers in AD [64]. However, these putative mechanisms are speculative and experimental mechanistic studies are required to extend the findings of the present study.

We acknowledge several limitations to the current study. First, the current focus was on serum proteins which may differ from those within cells and tissues and should also be explored for disease associations. Second, European participants accounted for the vast majority of the current cohort and findings may not be generalizable to populations with different ethnicities. Thirdly, a cis-pQTL coding variant might change a protein’s amino acid sequence without necessarily impacting its function or level. Equating sequence alterations with functional changes could lead to incorrect conclusions. Caution should be exercised in the interpretation of these results. Last, publicly available datasets were used and represent data resources for target identification which are not new, although novel insights and perspectives may be drawn from them.

## Conclusion

A MR analysis was conducted to explore the proteomic pathogenesis of allergic disease. Examples of Tier 1 and 2 protein targets that are promising drug target candidates were STAT6, TNFRSF6B, IL1RL2, and IL6R for AA; CRAT, TNFRSF6B, ERBB3, IL6R, MMP12, ICAM1 and IL1RL2 for AD, and ICAM1, IL1RL2, MANF, STAT6 and TNFSF8 for AR. These proteins may influence the development of allergic diseases and expose new diagnostic and therapeutic targets. Further experiments are required to validate the current findings regarding proteinaceous allergic disease markers.

### Supplementary Information

Supplementary Material 1.

Supplementary Material 2.

## Data Availability

All analyses were conducted using publicly available data. The data that support this study are openly available in UK Biobank at https://www.ukbiobank.ac.uk/, and FinnGen, at https://www.finngen.fi/en. Code Availability: the analysis code in R is available on request.
