# Supplementary material for: Identification of plasma protein markers of allergic disease risk: a mendelian randomization approach to proteomic analysis
Source: BMC Genomics. 2024 May 22;25:503. doi: 10.1186/s12864-024-10412-0 (PMC11110418; doi:10.1186/s12864-024-10412-0)
Supplement: Supplementary file 2 — Supplementary Material 2. [file 12864_2024_10412_MOESM2_ESM.docx]

**Supplementary Figure 1.** Forest plot showing MR analysis results for plasma proteins and allergic asthma in the discovery and replication analysis, complemented by results from subsequent meta-analyses.

**Supplementary Figure 2.** Forest plot showing MR analysis results for plasma proteins and atopic dermatitis in the discovery and replication analysis, complemented by results from subsequent meta-analyses.

**Supplementary Figure 3.** Forest plot showing MR analysis results for plasma proteins and allergic rhinitis in the discovery and replication analysis, complemented by results from subsequent meta-analyses.

**Supplementary Figure 4.** Colocalization analysis of the identified plasma proteins and allergic asthma. Red dots indicate SNPs exhibiting significant combined P-values in both the protein GWAS and disease GWAS analyses. (A) APOE; (B) GALK1; (C) IL1RL2; (D) IL6R; (E) LRRC32; (F) MAX; (G) NPNT; (H) PILRA; (I) PRSS8; (J) STAT6; (K) TNFRSF6B; (L) VTA1.

**Supplementary Figure 5.** Colocalization analysis of the identified plasma proteins and atopic dermatitis. Red dots indicate SNPs exhibiting significant combined P-values in both the protein GWAS and disease GWAS analyses. (A) CRAT; (B) ERBB3; (C) IL6R; (D) LRRC32; (E) MMP12; (F) TNFRSF6B.

**Supplementary Figure 6.** Colocalization analysis of the identified plasma proteins and allergic rhinitis. Red dots indicate SNPs exhibiting significant combined P-values in both the protein GWAS and disease GWAS analyses. (A) ERBB3; (B) FCRLB; (C) ICAM; (D) 1IL1R; (E) 1IL1RL2; (F) IL7R; (G) MANF; (H) PILRA; (I) STAT6; (J) TNFSF8.

**Supplementary Figure 7.** Manhattan plot illustrating the external validation analyses of identified plasma proteins for allergic diseases. (A) PWAS results of allergic asthma; (B) PWAS results of atopic dermatitis; (C) PWAS results of allergic rhinitis; (D) TWAS results of allergic asthma; (E) TWAS results of atopic dermatitis; (F) TWAS results of allergic rhinitis;

**Supplementary Figure 8.** Protein-protein interaction network between identified proteins linked to allergic asthma and known drug targets from DrugBank database. Circles represent proteins, and the lines between them indicate significant associations, with the thickness of the lines denoting the strength of these associations.

**Supplementary Figure 9.** Protein-protein interaction network between identified proteins linked to atopic dermatitis and known drug targets from DrugBank database. Circles represent proteins, and the lines between them indicate significant associations, with the thickness of the lines denoting the strength of these associations.

**Supplementary Figure 10.** Protein-protein interaction network between identified proteins linked to allergic rhinitis and known drug targets from DrugBank database. Circles represent proteins, and the lines between them indicate significant associations, with the thickness of the lines denoting the strength of these associations.

**Supplementary Figure 11.** Bar plots and bubble plots of the top 20 pathways for the enrichment analyses for identified plasma proteins and known drug targets from DrugBank database with allergic diseases. (A) GO enrichment analysis for allergic asthma; (B) GO enrichment analysis for atopic dermatitis; (C) GO enrichment analysis for allergic rhinitis; (D) KEGG enrichment analysis for allergic asthma; (E) KEGG enrichment analysis for atopic dermatitis; (F) KEGG enrichment analysis for allergic rhinitis;


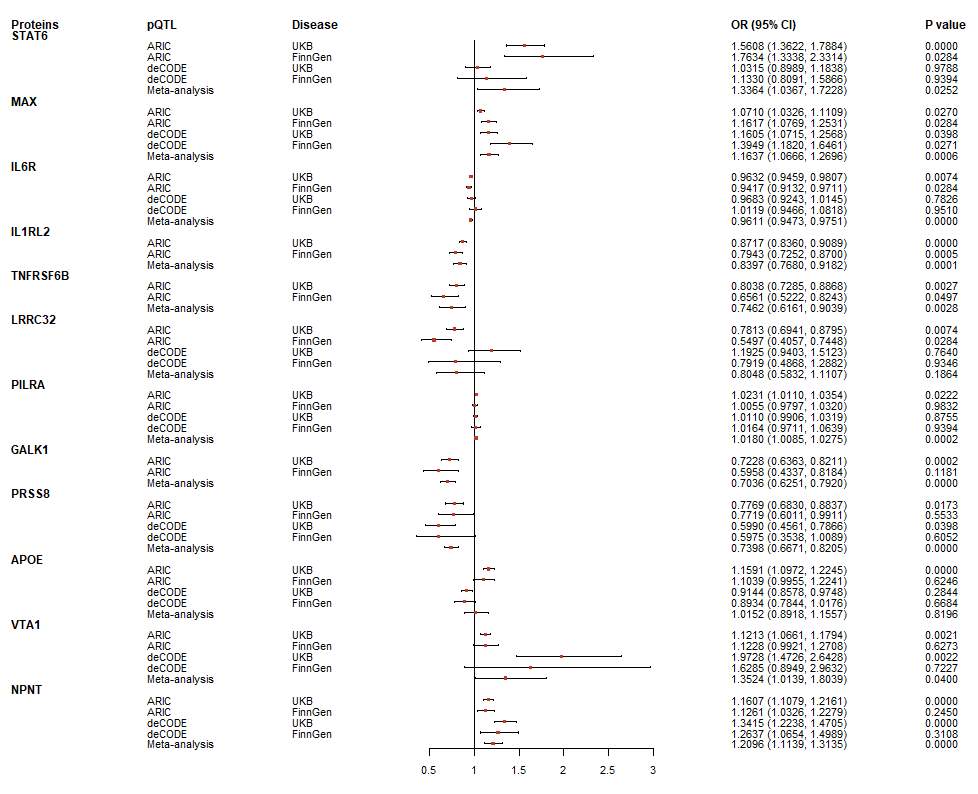


**Supplementary Figure 1.** Forest plot showing MR analysis results for plasma proteins and allergic asthma in the discovery and replication analysis, complemented by results from subsequent meta-analyses.


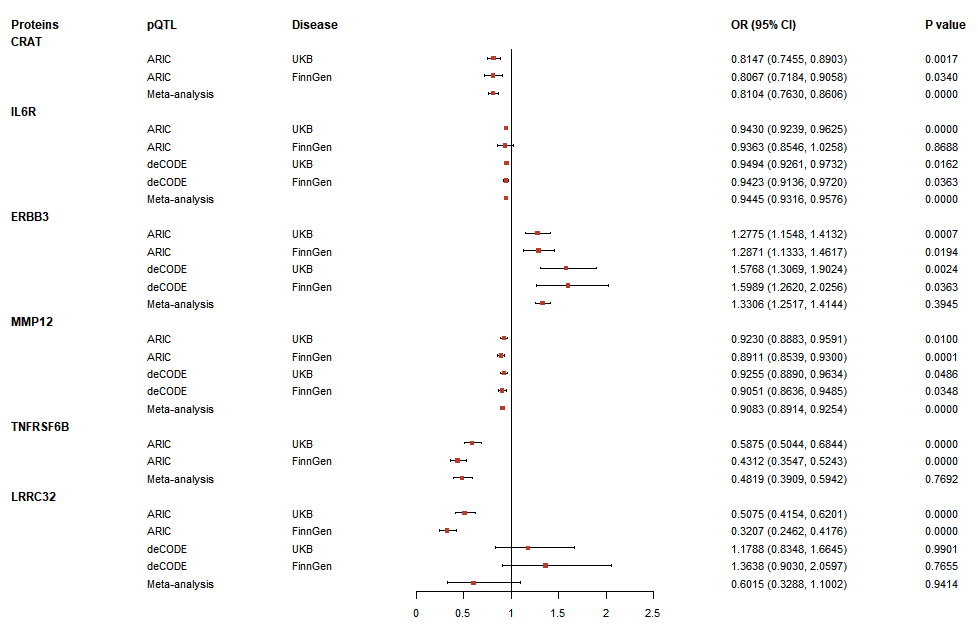


**Supplementary Figure 2.** Forest plot showing MR analysis results for plasma proteins and atopic dermatitis in the discovery and replication analysis, complemented by results from subsequent meta-analyses.


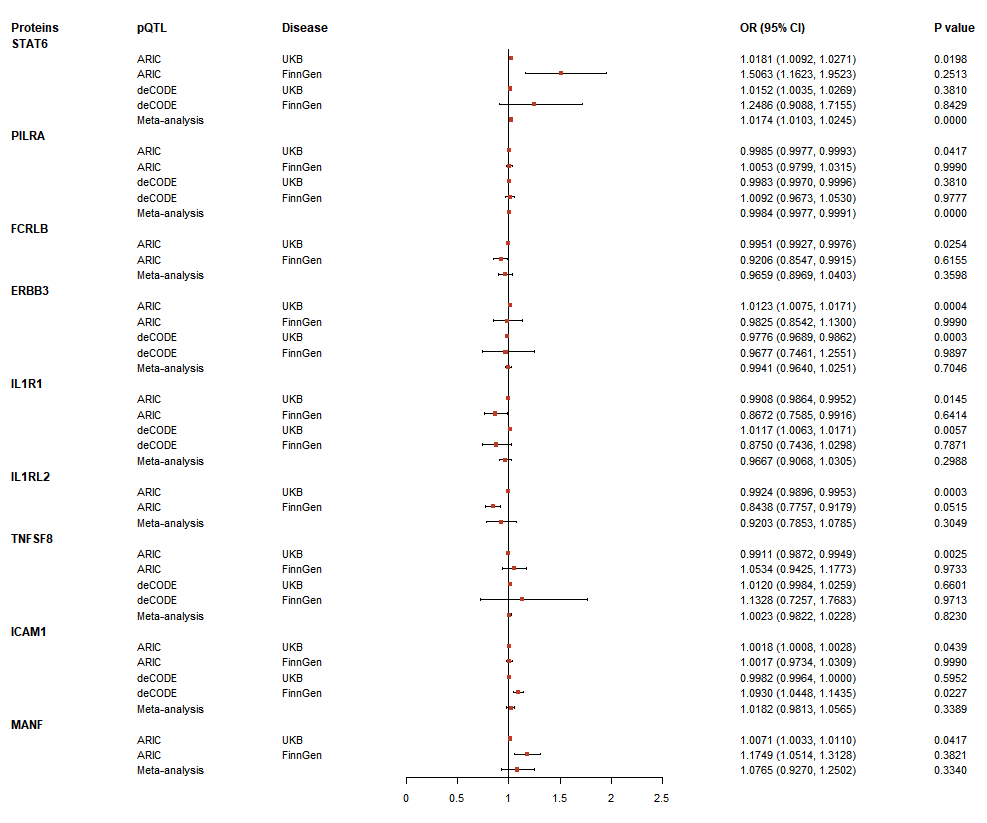


**Supplementary Figure 3.** Forest plot showing MR analysis results for plasma proteins and allergic rhinitis in the discovery and replication analysis, complemented by results from subsequent meta-analyses.

_
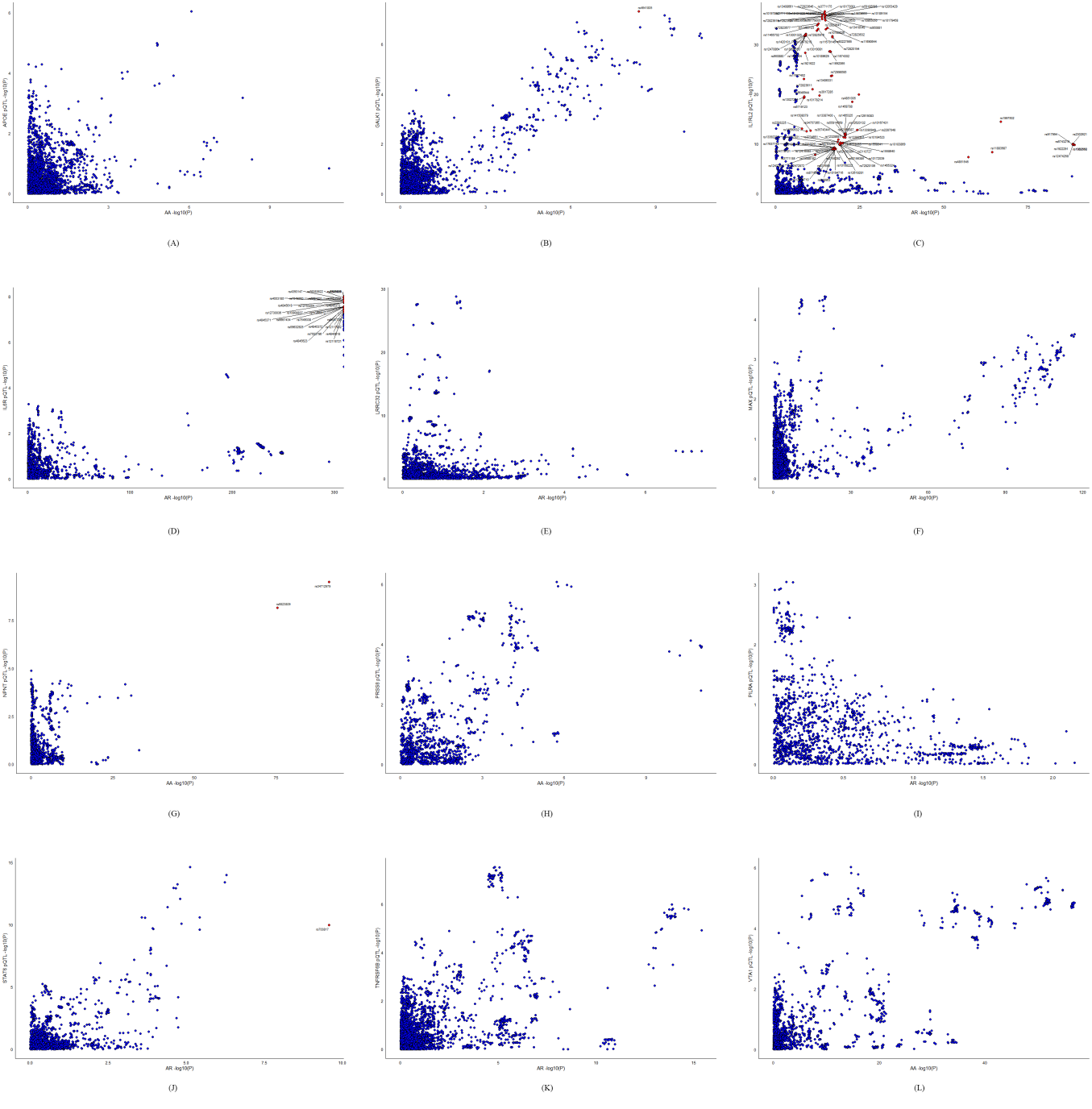
_

**Supplementary Figure 4.** Colocalization analysis of the identified plasma proteins and allergic asthma. Red dots indicate SNPs exhibiting significant combined P-values in both the protein GWAS and disease GWAS analyses. (A) APOE; (B) GALK1; (C) IL1RL2; (D) IL6R; (E) LRRC32; (F) MAX; (G) NPNT; (H) PILRA; (I) PRSS8; (J) STAT6; (K) TNFRSF6B; (L) VTA1.

**
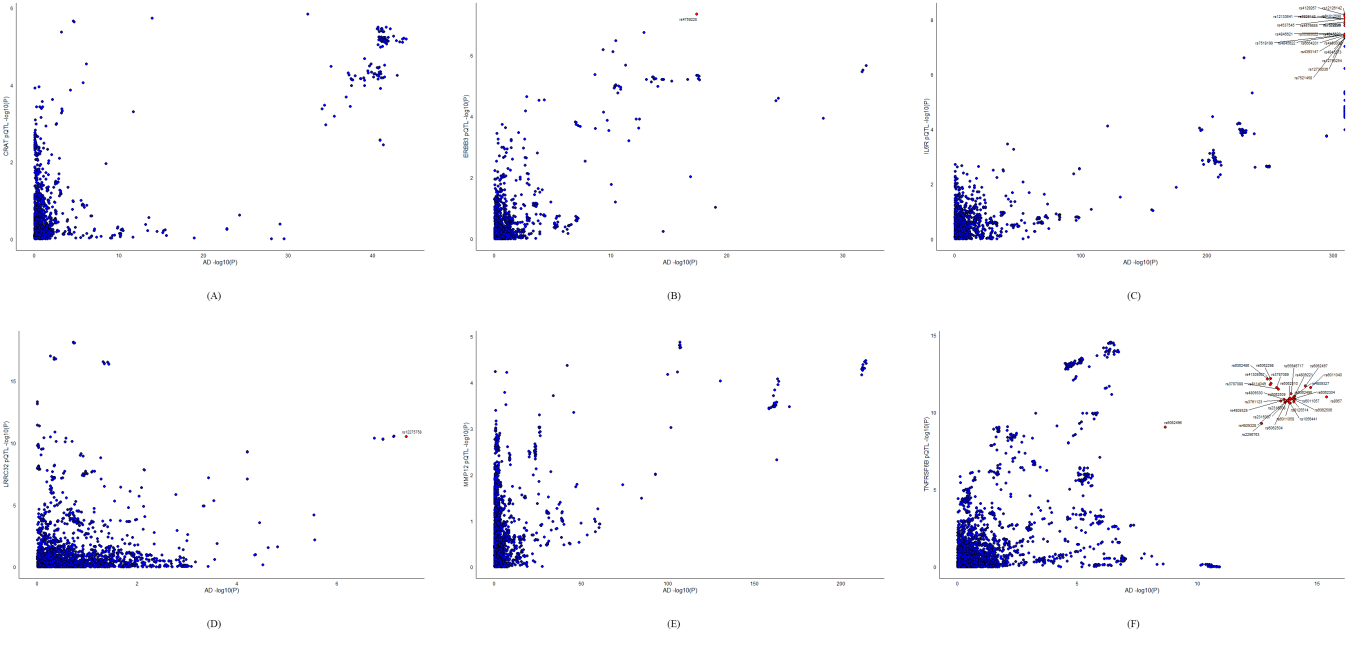
**

**Supplementary Figure 5.** Colocalization analysis of the identified plasma proteins and atopic dermatitis. Red dots indicate SNPs exhibiting significant combined P-values in both the protein GWAS and disease GWAS analyses. (A) CRAT; (B) ERBB3; (C) IL6R; (D) LRRC32; (E) MMP12; (F) TNFRSF6B.


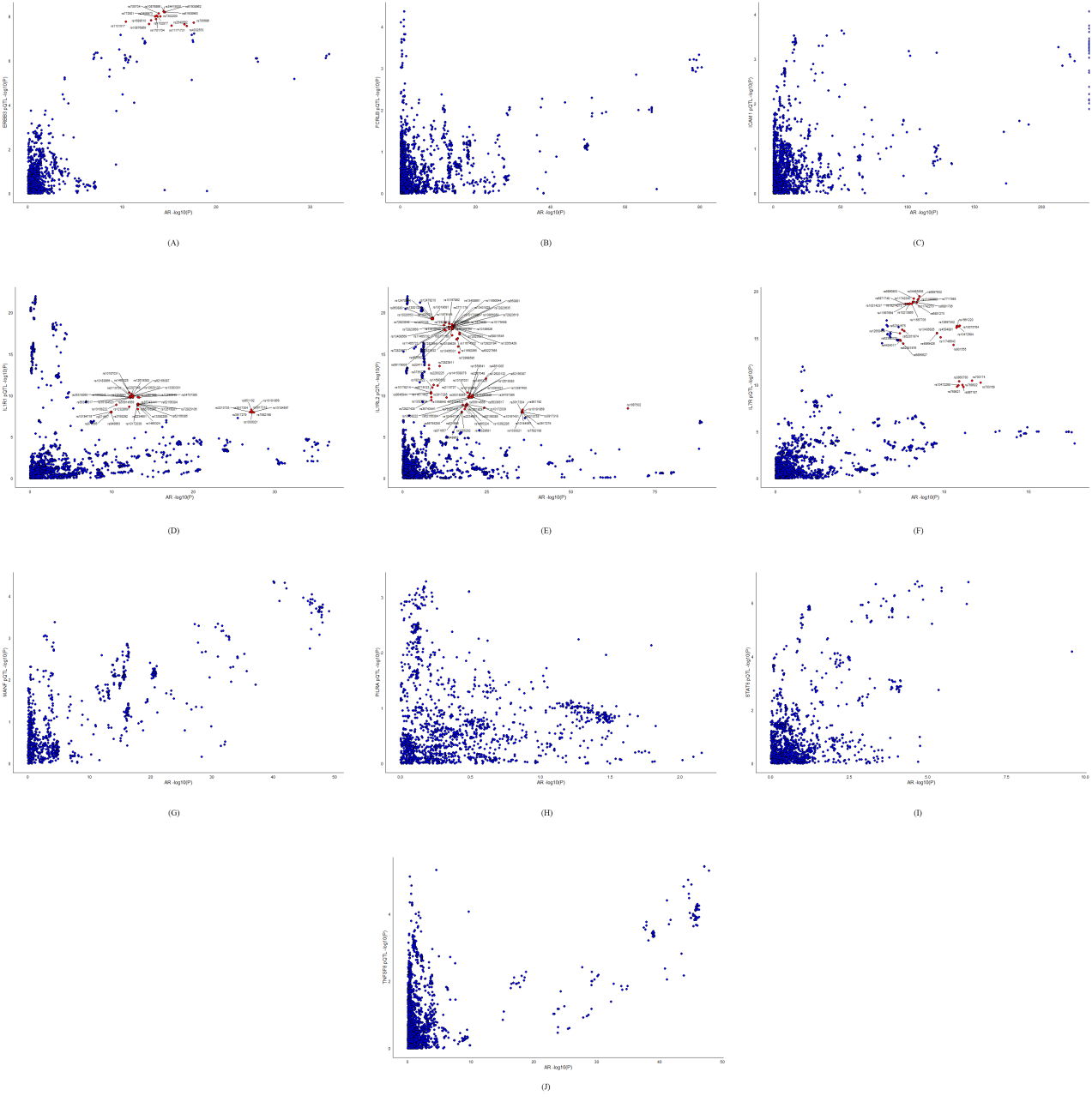


**Supplementary Figure 6.** Colocalization analysis of the identified plasma proteins and allergic rhinitis. Red dots indicate SNPs exhibiting significant combined P-values in both the protein GWAS and disease GWAS analyses. (A) ERBB3; (B) FCRLB; (C) ICAM; (D) 1IL1R; (E) 1IL1RL2; (F) IL7R; (G) MANF; (H) PILRA; (I) STAT6; (J) TNFSF8.


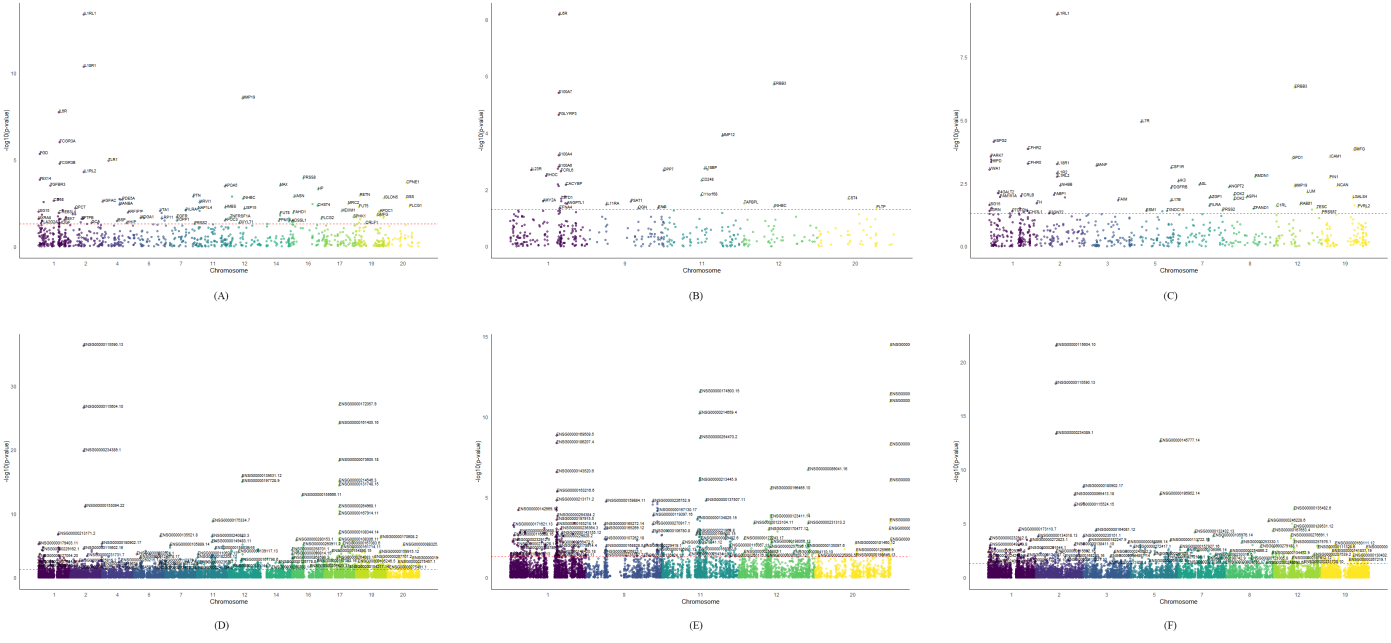
**Supplementary Figure 7.** Manhattan plot illustrating the external validation analyses of identified plasma proteins for allergic diseases. (A) PWAS results of allergic asthma; (B) PWAS results of atopic dermatitis; (C) PWAS results of allergic rhinitis; (D) TWAS results of allergic asthma; (E) TWAS results of atopic dermatitis; (F) TWAS results of allergic rhinitis;


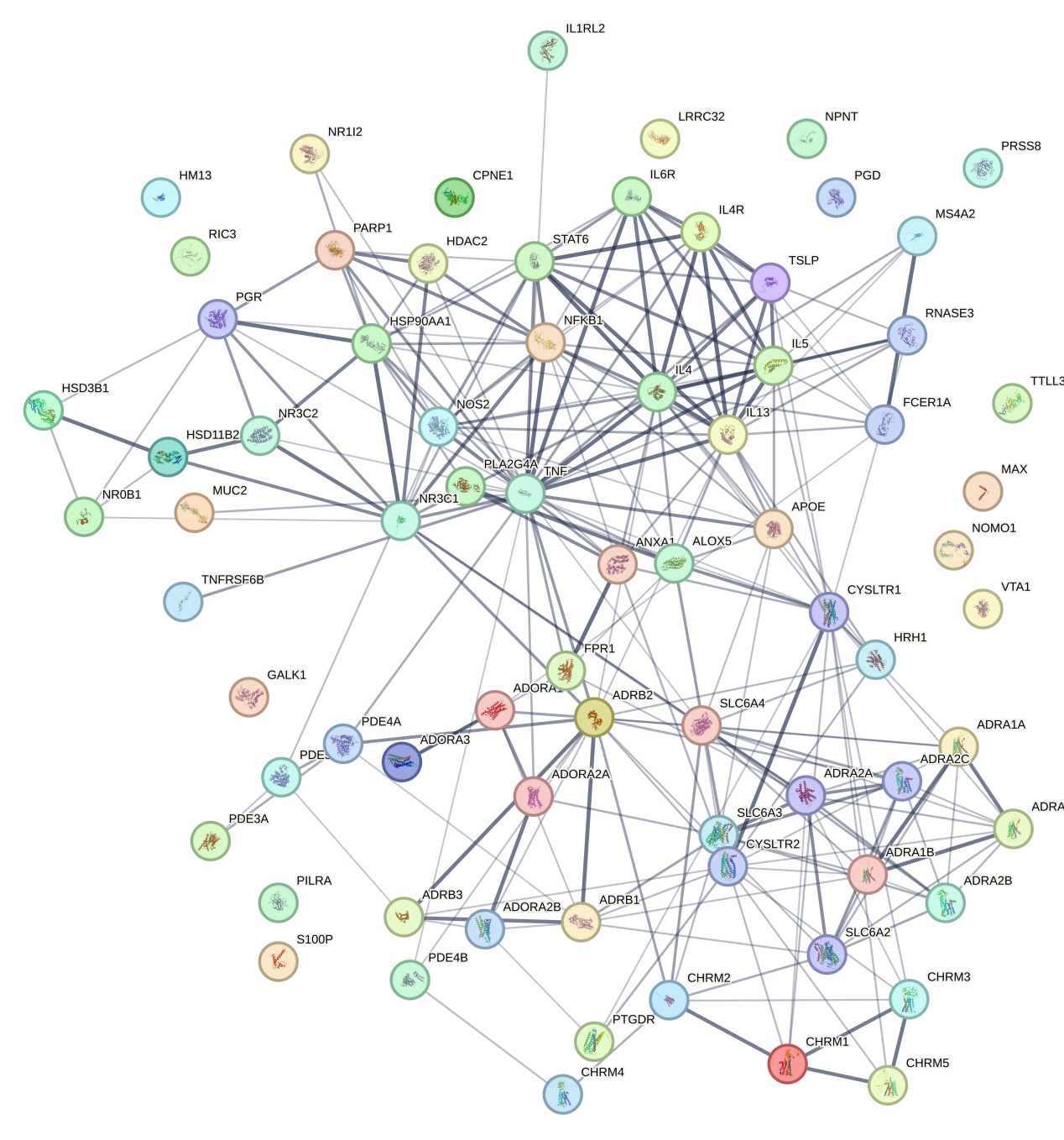


**Supplementary Figure 8.** Protein-protein interaction network between identified proteins linked to allergic asthma and known drug targets from DrugBank database. Circles represent proteins, and the lines between them indicate significant associations, with the thickness of the lines denoting the strength of these associations.


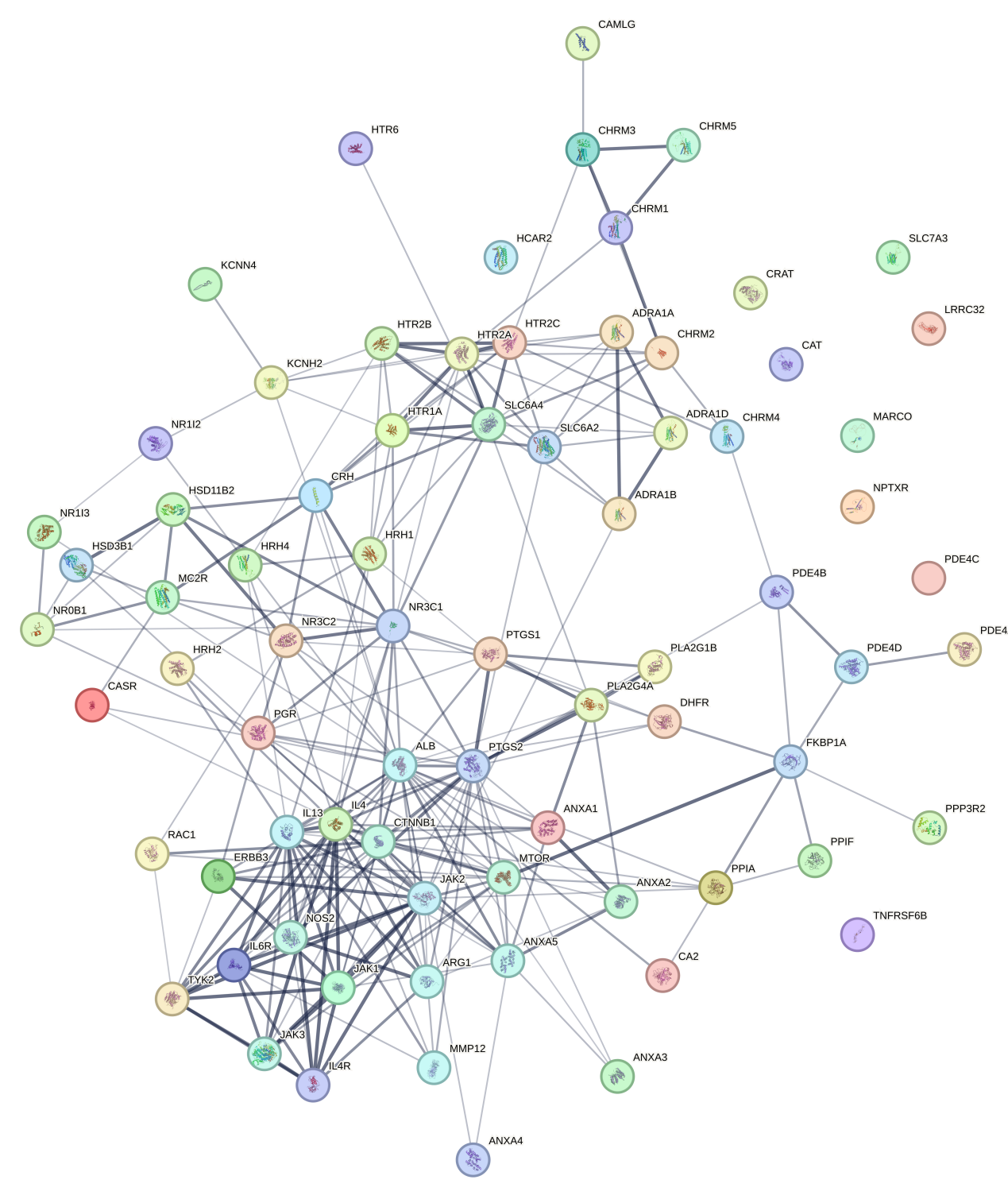


**Supplementary Figure 9.** Protein-protein interaction network between identified proteins linked to atopic dermatitis and known drug targets from DrugBank database. Circles represent proteins, and the lines between them indicate significant associations, with the thickness of the lines denoting the strength of these associations.


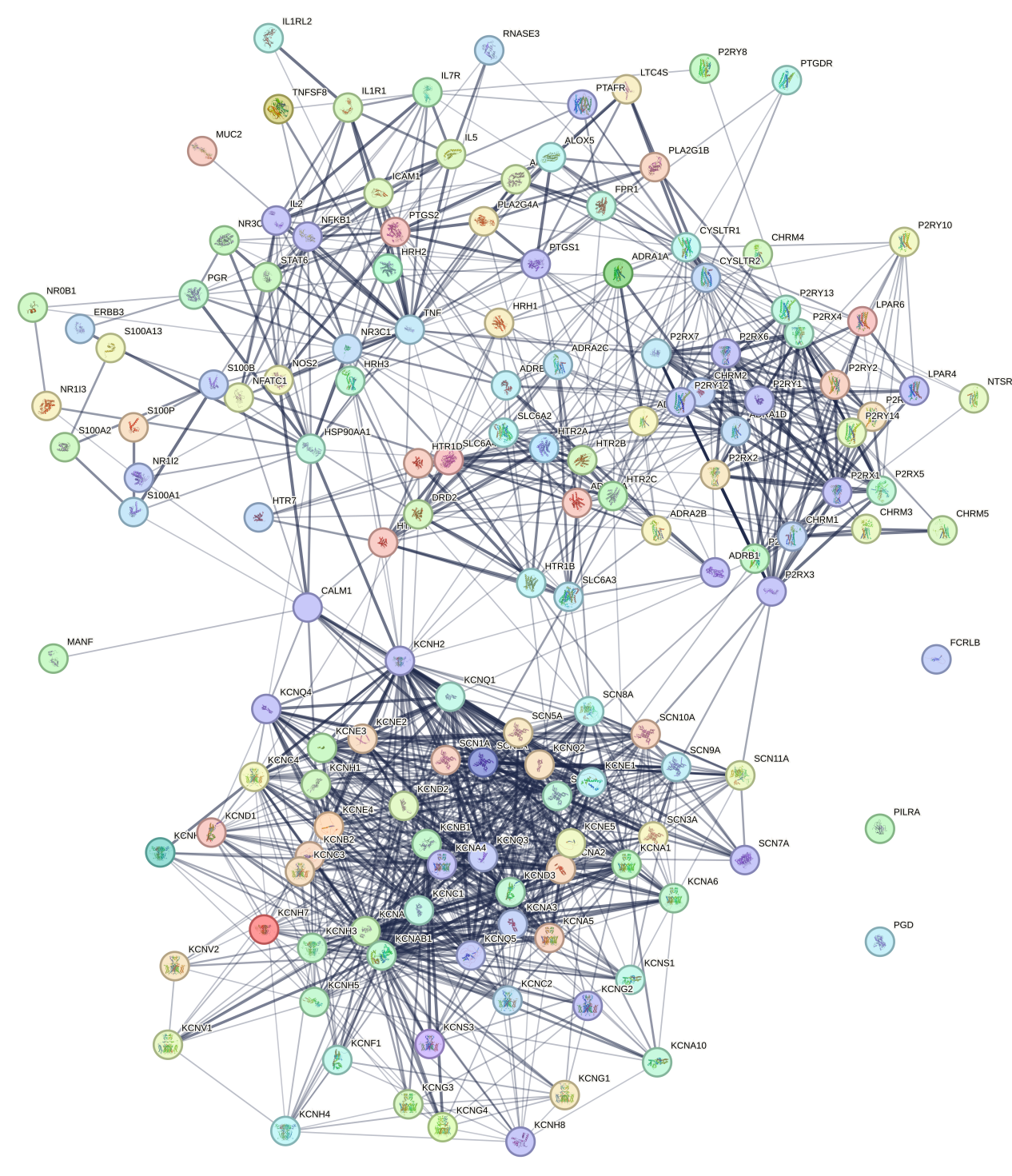


**Supplementary Figure 10.** Protein-protein interaction network between identified proteins linked to allergic rhinitis and known drug targets from DrugBank database. Circles represent proteins, and the lines between them indicate significant associations, with the thickness of the lines denoting the strength of these associations.


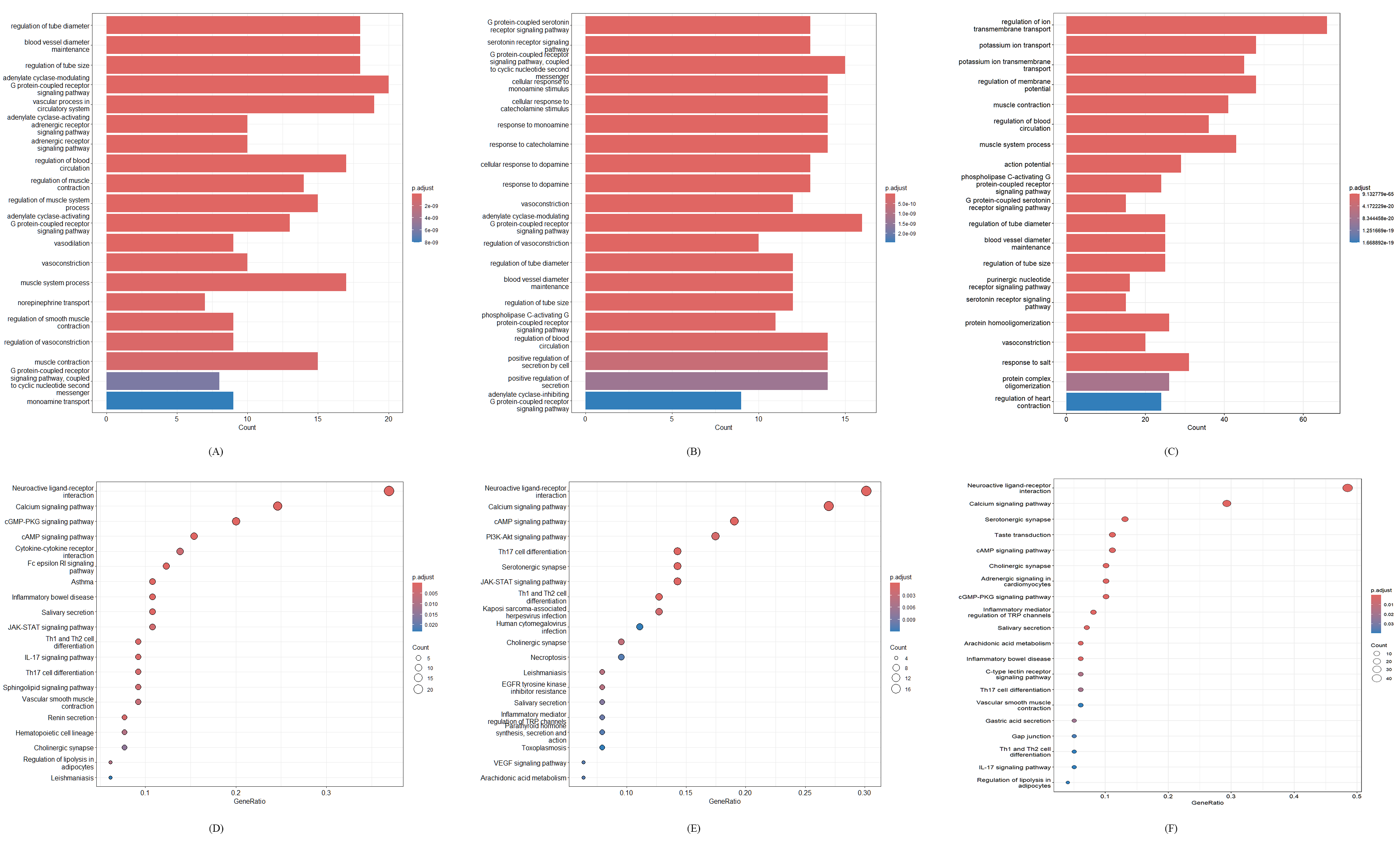


**Supplementary Figure 11.** Bar plots and bubble plots of the top 20 pathways for the enrichment analyses for identified plasma proteins and known drug targets from DrugBank database with allergic diseases. (A) GO enrichment analysis for allergic asthma; (B) GO enrichment analysis for atopic dermatitis; (C) GO enrichment analysis for allergic rhinitis; (D) KEGG enrichment analysis for allergic asthma; (E) KEGG enrichment analysis for atopic dermatitis; (F) KEGG enrichment analysis for allergic rhinitis;
